# Breath analysis by gas chromatography-mass spectrometry and electronic nose to screen for pleural mesothelioma: a cross-sectional case-control study

**DOI:** 10.18632/oncotarget.21335

**Published:** 2017-09-27

**Authors:** Kevin Lamote, Paul Brinkman, Lore Vandermeersch, Matthijs Vynck, Peter J. Sterk, Herman Van Langenhove, Olivier Thas, Joris Van Cleemput, Kristiaan Nackaerts, Jan P. van Meerbeeck

**Affiliations:** ^1^ Department of Respiratory Medicine, Ghent University Hospital, Ghent, Belgium; ^2^ Department of Internal Medicine, Ghent University, Ghent, Belgium; ^3^ Department of Respiratory Medicine, Academic Medical Centre, University of Amsterdam, Amsterdam, The Netherlands; ^4^ Department of Sustainable Organic Chemistry and Technology, EnVOC Research Group, Ghent University, Ghent, Belgium; ^5^ Department of Mathematical Modelling, Statistics and Bio-Informatics, Ghent University, Ghent, Belgium; ^6^ Occupational Health Service, Eternit N.V., Kapelle-op-den-Bos, Belgium; ^7^ Department of Respiratory Diseases, KU Leuven, University Hospitals Leuven, Leuven, Belgium; ^8^ Thoracic Oncology/MOCA, Antwerp University Hospital, Edegem, Belgium

**Keywords:** mesothelioma, breath tests, volatile organic compounds, gas chromatography-mass spectrometry, electronic nose

## Abstract

**Rationale:**

Malignant pleural mesothelioma (MPM) is mainly caused by previous exposure to asbestos fibers and has a poor prognosis. Due to a long latency period between exposure and diagnosis, MPM incidence is expected to peak between 2020-2025. Screening of asbestos-exposed individuals is believed to improve early detection and hence, MPM management. Recent developments focus on breath analysis for screening since breath contains volatile organic compounds (VOCs) which reflect the cell’s metabolism.

**Objectives:**

The goal of this cross-sectional, case-control study is to identify VOCs in exhaled breath of MPM patients with gas chromatography-mass spectrometry (GC-MS) and to assess breath analysis to screen for MPM using an electronic nose (eNose).

**Methods:**

Breath and background samples were taken from 64 subjects: 16 healthy controls (HC), 19 asymptomatic former asbestos-exposed (AEx) individuals, 15 patients with benign asbestos-related diseases (ARD) and 14 MPM patients. Samples were analyzed with both GC-MS and eNose.

**Results:**

Using GC-MS, AEx individuals were discriminated from MPM patients with 97% accuracy, with diethyl ether, limonene, nonanal, methylcyclopentane and cyclohexane as important VOCs. This was validated by eNose analysis. MPM patients were discriminated from AEx+ARD participants by GC-MS and eNose with 94% and 74% accuracy, respectively. The sensitivity, specificity, positive and negative predictive values were 100%, 91%, 82%, 100% for GC-MS and 82%, 55%, 82%, 55% for eNose, respectively.

**Conclusion:**

This study shows accurate discrimination of patients with MPM from asymptomatic asbestos-exposed persons at risk by GC-MS and eNose analysis of exhaled VOCs and provides proof-of-principle of breath analysis for MPM screening**.**

## INTRODUCTION

Malignant pleural mesothelioma (MPM) is an aggressive tumor originating from the pleural lining of the thorax and is causally associated with previous asbestos exposure [1, 2]. Despite a ban on asbestos use in the entire European Union in 2005, asbestos is still being produced and consumed in several countries in need for industrial growth. Together with a long average latency period of 40-50 years between first asbestos exposure and MPM diagnosis, this indicates that MPM incidence will further increase [3]. With a 5-year survival rate below 5%, prognosis remains poor, stressing the need for an earlier diagnosis by screening. Serum biomarkers have not proven to be useful for the screening and diagnosis of MPM [4, 5]. Therefore, recent research focused on breath analysis [6]. Breath contains volatile organic compounds (VOCs) which arise from the body’s (patho)physiological processes and have demonstrated to be useful in the detection of asthma, COPD, and tumors [7-12].

Asbestos fibers are known to initiate oxidative stress at the mesothelium [13], inducing lipid peroxidation of the mesothelial cell wall, releasing VOCs, and mutagenic DNA lesions. Furthermore, asbestos fibers activate the NF-κB pathway and promote cell survival which contributes to MPM development [14]. VOCs enter the bloodstream, are transported to the lungs where they enter the alveoli through the gas exchange mechanisms and finally are exhaled. Few studies have addressed the use of VOCs for MPM detection. One study analyzed breath samples from 13 MPM patients, 13 occupationally asbestos-exposed persons, and 13 healthy non-exposed controls using gas chromatography-mass spectrometry (GC-MS) [15]. Cyclohexane allowed to discriminate MPM patients with 97.4% accuracy. Two studies used pattern recognition of exhaled VOCs by cross-reactive sensor technology (electronic nose: eNose) to compare breath samples of the same 3 groups. Dragonieri *et al.* [16] and Chapman *et al.* [17] distinguished MPM patients from controls with 92.3% and 90% sensitivity, respectively. Recently, our research group was able to discriminate 23 MPM patients from 22 asymptomatic occupationally asbestos-exposed persons and 21 healthy non-exposed controls with 87% sensitivity and 70% specificity using multicapillary column-ion mobility spectrometry (MCC/IMS) [18]. Nevertheless, these studies have not been replicated nor has GC-MS been directly validated against eNose.

Since MPM is linked to asbestos exposure and oxidative stress, we hypothesize that VOCs and VOC patterns will differ between MPM patients, persons occupationally exposed to asbestos, and those unexposed. To that end, we aimed to identify discriminating VOCs by GC-MS and assess the between-group comparisons with eNose in order to provide the proof-of-principle of screening for MPM by breath analysis.

## RESULTS

### Patient characteristics

Sixty-four participants were included: 14 treatment-naïve MPM patients, 15 patients with benign asbestos-related diseases (ARD), 19 AEx persons, and 16 HC individuals (Table 1).

**Table 1:** Patient characteristics

	HC	AEx	ARD	MPM	*p-value*
**N**	16	19	15	14	
**Gender**					
*Male*	15 (93.8%)	19 (100%)	14 (93.3%)	11 (78.6%)	*0.173*^*a*^
*Female*	1 (6.3%)	0 (0.0%)	1 (6.7%)	3 (22.4%)	
**Age**	56 (52.5 – 59.4)	50 (49.6 – 53.2)	60 (58.3 – 63.8)	69 (65.7 – 73.6)	*<0.001*^*b*^
**Smoke status**					
*Current*	0 (0.0%)	6 (31.6%)	1 (6.7%)	1 (7.1%)	
*EX*	8 (50.0%)	7 (36.8%)	5 (33.3%)	9 (64.3%)	*0.079*^*a*^
*Never*	8 (50.0%)	6 (31.6%)	9 (60.0%)	4 (28.6%)	
**Pack years**	0.3 (0.0 - 6.1)	9.0 (0.0 - 36.0)	0 (0.0 – 10.5)	7 (0.0 – 30.0)	*0.106*^*b*^
**BMI (kg/m**^2^**)**	27 (23.4 – 29.3)	27 (25.4 – 28.4)	27 (24.5 – 32.8)	26 (23.9 – 27.1)	*0.529*^*b*^

^a^Fisher’s exact test.

^b^Non-parametric Kruskal-Wallis test.

AEx: asymptomatic former asbestos-exposed controls. ARD: patients with benign asbestos related diseases. BMI: body mass index. HC: healthy controls. MPM: malignant pleural mesothelioma.

MPM patients were significantly older than the other groups; AEx persons were the youngest. No significant differences were found in smoking status, pack years or BMI between the groups although we observed a trend with AEx persons having have more current smokers. Among the ARD patients, 14 (93%) had pleural plaques and 1 (7%) had asbestosis.

### GC-MS analysis

**Figure 1:**
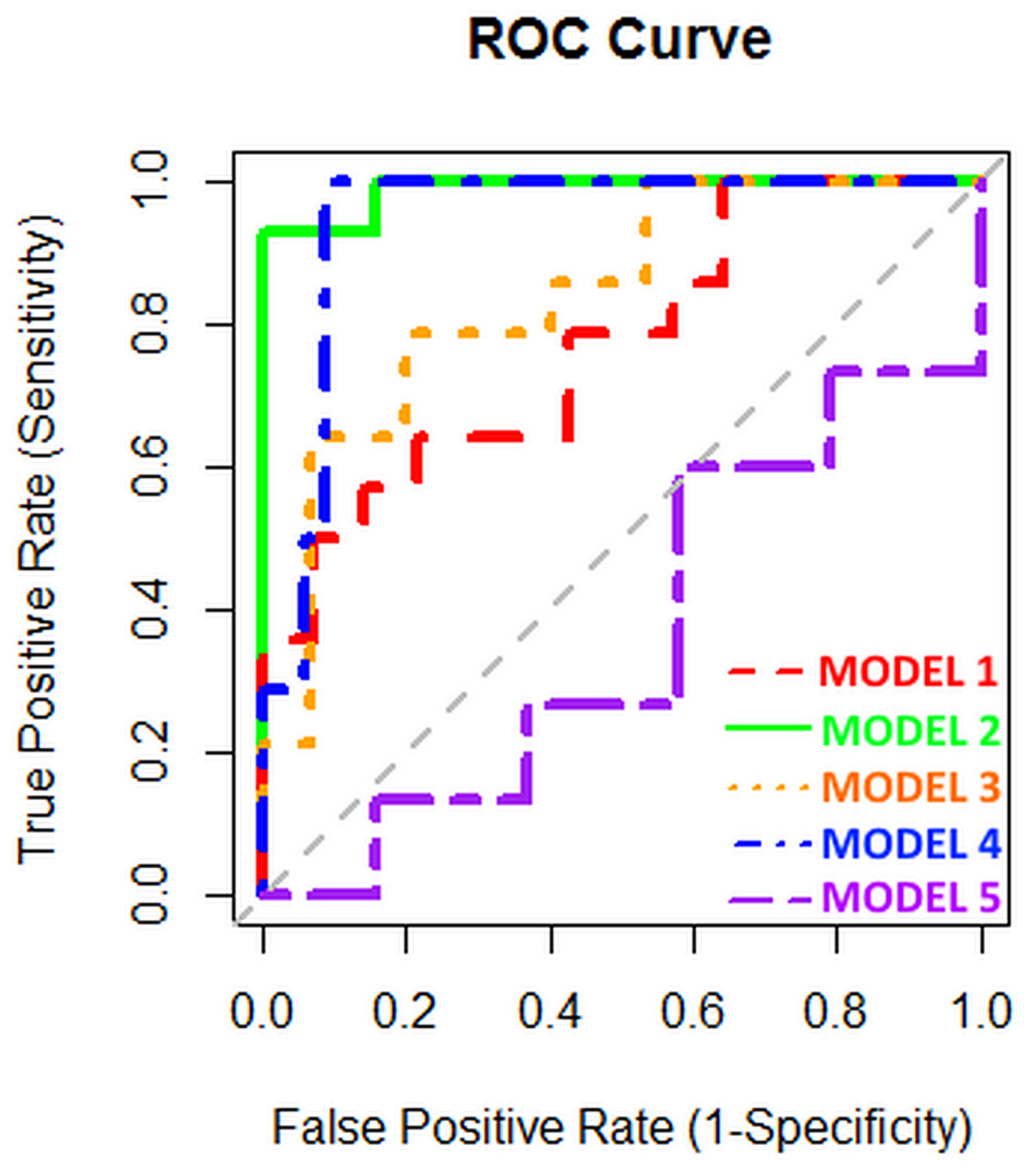
ROC curves of the different models based upon GC-MS analysis

In total, 14 MPM patients, 19 AEx subjects, 15 ARD patients and 14 HC controls gave a breath sample for GC-MS analysis. We analyzed 5 different models (Table 2, Figure 1): MPM vs. HC (model 1), MPM vs. AEx (model 2), MPM vs. ARD (model 3), MPM vs. AEx+ARD (model 4) and ARD vs. AEx (model 5). Model 1 showed a diagnostic accuracy of 71% (52.9%-85.7%). The AUC_ROC_ was 0.770.

**Table 2:** Model characteristics from GC-MS data

	Model 1	Model 2	Model 3	Model 4	Model 5
*Cases vs controls*	*MPM vs HC*	*MPM vs AEx*	*MPM vs ARD*	*MPM vs AEx+ARD*	*AEx vs ARD*
*N*	*14 vs 14*	*14 vs 19*	*14 vs 15*	*14 vs 34*	*19 vs 15*
Sensitivity	64.3% (37.6%-85.6%)	92.9% (69.5%-99.6%)	78.6% (52.1%-94.2%)	100% (80.7%-100%)	60.0% (34.6%-81.9%)
Specificity	78.6% (52.1%-94.2%)	100% (85.4%-100%)	80.0% (54.7%-94.6%)	91.2% (77.9%-97.7%)	42.1% (21.9%-64.6%)
PPV	75.0% (45.9%-93.2%)	100% (79.4%-100%)	78.6% (52.1%-94.2%)	82.4% (59.2%-95.3%)	45.0% (24.7%-66.7%)
NPV	68.8% (43.7%-87.5%)	95.0% (77.8%-99.7%)	80.0% (54.7%-94.6%)	100% (90.8%-100%)	57.1% (31.2%-80.4%)
Accuracy	71.4% (52.9%-85.7%)	97.0% (86.0%-99.8%)	79.3% (61.9%-91.2%)	93.8% (84.0%-98.4%)	50.0% (33.6%-66.4%)
AUC_ROC_	0.770 (0.577-0.923)^#^	0.989 (0.955 – 1.000)^#^	0.838 (0.671-0.962)^#^	0.943 (0.866-1.000)^#^	0.365 (0.435-0.818)
VOCs (≥50% of times selected)	Nonane VOC I_*K*_ 1349 Propylbenzene Benzonitrile Isoprene Limonene 3-methylpentane 1,3-dichlorobenzene	Ethanol Diethyl ether 2-ethyl-1-hexanol Limonene Nonanal 2-methyl-1-propanol Methylcyclopentane Cyclohexane 1,2,4-trichlorobenzene Naphtalene VOC I_*K*_ 679 Phenol Chloroform Linalool Furfural VOC I_*K*_ 1287 Bromobenzene	VOC I_*K*_ 931 VOC I_*K*_ 1493 Beta-pinene Diethyl ether Limonene Hexane 1,2-dichlorobenzene	Ethanol Diethyl ether Isothiocyanatocyclohexane VOC I_*K*_ 1233 VOC I_*K*_ 1287 VOC I_*K*_ 1309 1,2-dichlorobenzene n-Butylbenzene Methylbenzoate 1,2,3-trichlorobenzene Limonene Bromobenzene VOC I_*K*_ 1100 Tert-butylbenzene m/p-xylene 2,2,4-trimethylpentane Hexamethyldisiloxane VOC I_*K*_ 1493 VOC I_*K*_ 720	Limonene Isopropyl acetate 1,3,5-triisopropylbenzene Diethyl ether 3,7-dimethyl-3-octanol Trichloroethylene Dimethyl disulfide Ethanol Phenol Acetophenone 2-methyl-1-propanol 1-butanol Naphtalene VOC I_*K*_ 615 1-methylthio-1-propene Isothiocyanatocyclohexane Isopropyl benzene VOC I_*K*_ 566 VOC I_*K*_ 1111 Hexanal VOC I_*K*_ 767 Ethylbenzene VOC I_*K*_ 1349 1,3-dichlorobenzene Dimethylsulfide VOC I_*K*_ 1105 2-hexanone VOC I_*K*_ 732 Nonane 3-methylpentane n-Butylbenzene

AEx: asymptomatic former asbestos-exposed controls. ARD: patients with benign asbestos related diseases. AUC_ROC_: area under the receiver operator characteristic curve. HC: healthy controls. I_*K*_: Kováts retention index. MPM: malignant pleural mesothelioma. NPV: negative predictive value. PPV: positive predictive value. VOC: volatile organic compound. ^#^: AUC_ROC_ statistically different from 0.5.

Since asbestos-exposed individuals can have a lifetime risk of MPM up to 10%, [13] we examined if it was possible to discriminate AEx and ARD participants from MPM patients in view of using it as screening tool (models 2-4). Discriminating MPM from AEx persons was possible with 97% accuracy (86.0%-99.8%), 93% sensitivity, 100% specificity, 100% PPV, and 95% NPV. The AUC_ROC_ was 0.989. Discriminating MPM from ARD patients was possible with 79% accuracy (61.9%-91.2%), 79% sensitivity, 80% specificity, 79% PPV, and 80% NPV. The AUC_ROC_ was 0.838. By pooling ARD and AEx persons, we could discriminate MPM patients with 94% accuracy (84.0%-98.4%), 100% sensitivity, 91% specificity, 82% PPV, and 100% NPV. The AUC_ROC_ was 0.943.

The most frequently selected VOCs in these discriminations were diethyl ether, limonene, cyclohexane, nonanal, VOC I_*K*_ 1287 and isothiocyanatocyclohexane (Table 2, Supplementary Figures 1-2).

As negative control analysis, we tried to discriminate ARD patients from AEx persons (model 5). This was not possible, showing 50% accuracy (33.6%-66.4%) and an AUC_ROC_ of 0.365, even when important discriminators from the other models were included.

### eNose analysis

**Figure 2:**
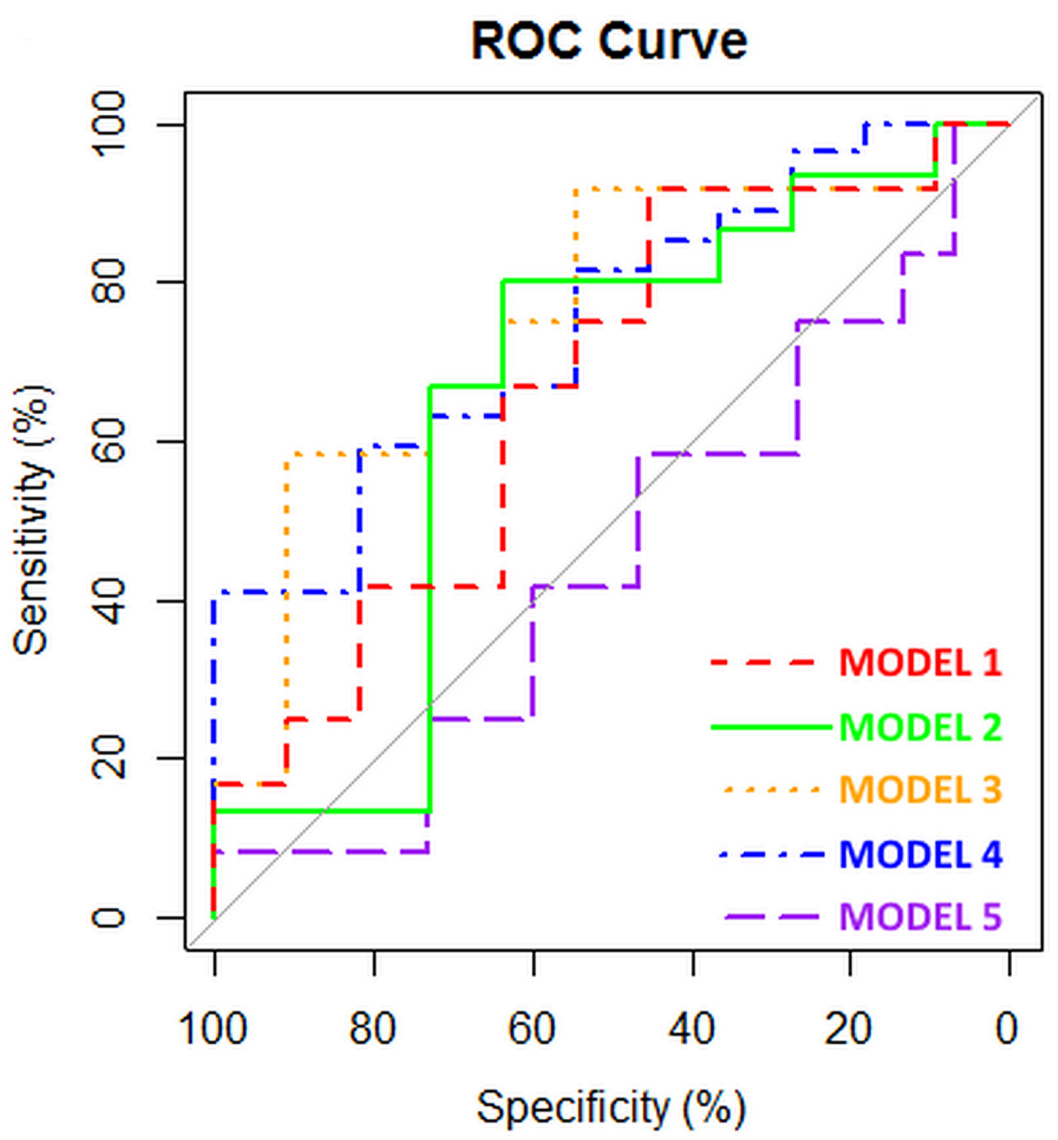
ROC curves of the different models based upon eNose analysis

In total, 11 MPM patients, 15 AEx subjects, 12 ARD patients and 12 HC controls gave a breath sample for eNose analysis. We analyzed the same 5 models as with GC-MS analysis (Table 3, Figure 2). We were able to discriminate MPM patients from HC controls (model 1) with 65% accuracy (44.5%-82.3%). The AUC_ROC_ was 0.667. Discriminating MPM from AEx persons was possible with 73% accuracy (53.9%-87.4%), 80% sensitivity, 64% specificity, 75% PPV, and 70% NPV. The AUC_ROC_ was 0.655.

**Table 3:** Model characteristics from eNose data

	Model 1	Model 2	Model 3	Model 4	Model 5
*Cases vs controls*	*MPM vs HC*	*MPM vs AEx*	*MPM vs ARD*	*MPM vs AEx+ARD*	*AEx vs ARD*
*N*	*11 vs 12*	*11 vs 15*	*11 vs 12*	*11 vs 27*	*15 vs 12*
Sensitivity	66.7% (37.7%-88.4%)	80.0% (54.7%-94.6%)	75.0% (45.9%-93.2%)	81.5% (63.7%-92.9%)	58.3% (30.3%-82.8%)
Specificity	63.6% (33.7%-87.2%)	63.6% (33.7%-87.2%)	63.6% (33.7%-87.2%)	54.5% (26.0%-81.0%)	46.7% (23.2%-71.3%)
PPV	66.7% (37.7%-88.4%)	75.0% (50.1%-91.5%)	69.2% (41.3%-89.4%)	81.5% (63.7%-92.9%)	46.7% (23.2%-71.3%)
NPV	63.6% (33.7%-87.2%)	70.0% (38.0%-91.7%)	70.0% (38.0%-91.7%)	54.5% (26.0%-81.0%)	58.3% (30.3%-82.8%)
Accuracy	65.2% (44.5%-82.3%)	73.1% (53.9%-87.4%)	69.6% (48.9%-85.6%)	73.7% (58.1%-85.8%)	51.9% (33.4%-70.0%)
AUC_ROC_	0.667 (0.434-0.900)	0.655 (0.416-0.893)	0.758 (0.548-0.967)^#^	0.747 (0.582-0.913)^#^	0.550 (0.322-0.778)

AEx: asymptomatic former asbestos-exposed controls. ARD: patients with benign asbestos related diseases. AUC_ROC_: area under the receiver operator characteristic curve. HC: healthy controls. MPM: malignant pleural mesothelioma. NA: not applicable. NPV: negative predictive value. PPV: positive predictive value. ^#^: AUC_ROC_ statistically different from 0.5.

MPM patients could be discriminated from ARD patients with 70% accuracy (48.9%-85.6%), 75% sensitivity, 64% specificity, 69% PPV, and 70% NPV. The AUC_ROC_ was 0.758. When ARD and AEx persons were pooled, we could discriminate MPM patients with 74% accuracy (58.1%-85.8%), 82% sensitivity, 55% specificity, 82% PPV, and 55% NPV. The AUC_ROC_ was 0.747.

Again, it was not possible to discriminate AEx persons from ARD patients, showing 52% accuracy (33.4%-70.0%) and an AUC_ROC_ of 0.550.

## DISCUSSION

In this cross-sectional study, breath analysis by GC-MS allows to discriminate with acceptable accuracy mesothelioma patients from healthy controls, patients with benign asbestos-related diseases, and asymptomatic individuals occupationally exposed to asbestos fibers in the past. This was replicated with an eNose. To our knowledge, this is the first time these discriminations are shown using multiple control groups and taking samples in parallel for GC-MS analysis and eNose replication by using previously validated methods [12]. Considering that occupationally asbestos-exposed persons have a lifetime increased risk for MPM and the latter is a lethal condition with a late-onset development, early detection is of utter importance to improve the disease’s management. Therefore, we examined whether it was possible to discriminate MPM patients from AEx and ARD persons. By GC-MS, we discriminated MPM from AEx and ARD persons with 97% and 79% accuracy, respectively. When both groups were pooled, the accuracy was 94%. Given the large sensitivity and NPV of these findings, the present study underlines the capacity of breath analysis as screening tool for persons at risk for MPM.

The most important VOCs selected in all of these GC-MS discriminations are nonanal, diethyl ether, limonene, methylcyclopentane, cyclohexane, a VOC with I_*K*_ 1287, and isothiocyanatocyclohexane. Furthermore, AEx persons were not easily discriminated from ARD patients, even when including the VOCs that discriminated MPM from the at risk groups, serving as negative controls. This underlines their importance as breath biomarkers for the presence of MPM.

Our results confirm and extend the findings from previous studies [15-19]. Using GC-MS, de Gennaro *et al.* discriminated 13 MPM patients from 13 HC controls and 13 AEx persons with 97.4% accuracy using cyclopentane, cyclohexane, dodecane, dimethyl nonane, limonene and β-pinene [15]. The group showed cyclohexane to be an important MPM marker and cyclopentane as marker for asbestos-exposure. We also found cyclohexane and limonene to be important in the discrimination of MPM from AEx patients and β-pinene to discriminate ARD from MPM patients. Furthermore, we found diethyl ether and nonanal important discriminators of MPM from AEx and/or ARD patients. These compounds are also found discriminative for lung cancer and are likely to be associated with tumorigenesis [20-22]. This adds to the plausibility of the discriminating capacity of these compounds of MPM.

Furthermore, Cakir *et al.* discriminated ARD patients and HC controls using ion mobility spectrometry with 99.9% accuracy, 96% sensitivity, and 50% specificity based upon α-pinene and 4-ethyltoluol [19]. We did not find these compounds as important discriminators in our models, weakening their importance as markers for ARD.

The GC-MS findings were replicated by pattern recognition of VOCs obtained by the cross-reactive sensors from the eNose. We discriminated MPM patients from HC controls, AEx persons and ARD patients with 65%, 73%, and 70% accuracy, 67%, 80%, and 75% sensitivity and 64%, 64%, and 64% specificity, respectively. When AEx and ARD patients were pooled, MPM patients were discriminated with 74% accuracy, 82% sensitivity and 55% specificity. The finding that the discriminative capacity by the eNose were somewhat lower as compared to the GC-MS results is due to the smaller number of patients who gave a sample for eNose analysis and the fact that eNoses recognize the bulk of the breath rather than specific individual VOCs.

Although we are reaching the same conclusions, our results slightly differ from those previously reported. Using the Cyranose, Dragonieri *et al.* discriminated 13 MPM patients from 13 AEx controls with 92% sensitivity and 86% specificity and from 13 HC controls with 92% sensitivity and 69% specificity [16]. Furthermore, Chapman *et al.* discriminated 10 MPM patients from 32 HC controls with 90% sensitivity and 91% specificity and from 18 ARD patients with 90% sensitivity and 83% specificity [17]. This may not be unexpected because of the lower number of patients and the fact we merged the sensor defections of 4 devices as final eNose profile.

The strength of our study lies in the multiple groups design and the replication of the results between two essentially different technologies for molecular assessment in exhaled air. Nevertheless, we acknowledge our study has important limitations. First of all, the low number of included participants restricts its application in the whole population. However, our results are in line with previous research and stresses its potential as screening tool. Secondly, our patients and controls were not matched for age and a trend in difference in smoking status was found. This can be due to the long latency period between first asbestos-exposure and MPM diagnosis, delaying diagnosis to late stages in more elderly people. Furthermore, it is hard to find healthy controls without substantial comorbidities at matched age. The difference in smoking status can originate from the fact that asbestos workers were blue-collar workers; an industry known to have an increased incidence of smokers [23]. Nevertheless, since MPM development is independent of smoking, the impact of smoking status on our results is expected to be minimal and smoking-associated VOCs (benzene, 2,5-dimethylfuran, and toluene) were not selected in either model, underlining the independency. Thirdly, since this study retrospectively questioned the participants, we cannot exclude potential recall bias and, hence, we have no information about the duration and intensity of asbestos exposure in our patients and controls nor about the time of last exposure on the breath composition. Fourthly, although an inspiratory VOC-filter was used, it is possible that exogenous compounds could have contaminated the breath since inhaled VOCs can be stored for a long time in the body’s fat compartments [24], and the sampling and analysis materials used can also release compounds. Finally, despite a cross-sectional, case-control design, our study was not blinded and we took breath samples from participants with known diagnosis. The next step should be to perform a blinded, prospective, case-control, cohort study to assess the diagnostic features of the breath test.

Despite these limitations, we found MPM patients to be discriminated from the at risk groups with clinically relevant accuracy by both GC-MS and eNose analysis. The large sensitivity and NPV allows breath analysis to be used as screening tool for exclusion of disease in at risk persons and to enrich the fraction of individuals at risk for further screening. By doing so, not every asbestos-exposed person is subjected to repeated chest imaging procedures, which will help the monitoring of asbestos-exposed individuals to be more cost-effective and reduce the associated radiation exposure [25]. Future research should focus on the next step: validating our results in an independent, large, multicenter series with blinding of the investigator for the underlying disease, monitoring AEx persons over time and see how breath analysis can be used to screen for MPM. In addition, the VOCs should be compared and correlated to mesothelin and linked with the pathophysiology of MPM by comparing the VOCs in breath with those in the headspace of mesothelioma cell lines and pleural fluid. This will ultimately improve the specificity.

## MATERIALS AND METHODS

### Study design and participants

We performed a multicenter, cross-sectional, case-control study in 64 subjects. Fourteen MPM patients, fifteen patients with well-defined benign asbestos-related diseases (ARD), and sixteen healthy non-asbestos exposed (HC) controls were recruited in the three participating university hospitals. Nineteen asymptomatic former asbestos-exposed individuals with well-documented asbestos exposure, were recruited via the occupational health service of a Belgian fiber-cement factory that processed asbestos until 1997. Treatment-naïve MPM patients were included after diagnosis, confirmed by the Belgian Mesothelioma Pathology Panel. Exclusion criteria were the start of any anti-tumor treatment before breath sampling, and the presence of non-asbestos-related diseases in the control groups. Before inclusion, a recent CT scan or chest X-ray (<12 months) had to be present to confirm the medical condition. The study was approved by the Institutional Review Board of Ghent University Hospital (LONG 11-01; Belgian registration number B670201111954) and was conducted in accordance with the Helsinki Convention. Participants had to give their written informed consent and two questionnaires had to be completed: one to check if the participants met the inclusion criteria and one to collect data about demographics and past occupational asbestos exposure. For all patients, a detailed medical record had to be available.

### Breath sampling

Breath was sampled using a previously validated method [8, 12]. In short, participants breathed tidally with a nose clip into a 2-way non-rebreathing valve (Hans Rudolph 2700, Hans Rudolph, Kansas City, USA) with an inspiratory VOC-filter (A2, North Safety, Middelburg, NL) at the inlet side. After 5 minutes of tidal breathing, the participants inhaled maximally and the expiratory port was connected to a 10L Tedlar bag (SKC Inc., Eighty Four, PA, USA). Subsequently, the subjects exhaled a full vital capacity volume into the Tedlar bag which was closed afterwards. Within 10 minutes, the bag was connected to an external pump and 500ml of the breath sample was loaded onto a sorbent tube (3.5” long, 0.25” outer diameter) filled with 200mg Tenax^®^GR (35/60 mesh; Markes International Ltd., Llantrisant, UK) for GC-MS analysis at a flow rate of 100ml.min^-1^ for 5 minutes. Immediately afterwards, 500ml of the breath sample was loaded onto another Tenax^®^GR tube (Tenax^®^GR SS 6mm x 7” (CAMSCO, Houston, Texas, USA)) for eNose analysis at a flow rate of 250ml.min^-1^ for 2 minutes. The sampling tubes were tightened, packed in a glass jar, and sent out for central analysis.

### Gas chromatography – mass spectrometry (GC-MS) analysis

Prior to use, the Tenax^®^GR-tubes were conditioned for 1 hour at 300°C while being flushed with helium (50 ml.min^-1^). After conditioning but before sampling, the tubes were loaded with 10.7 ng toluene-d8 internal standard, by making a two-phase system and using a home-made injector system. After sampling, breath analytes were desorbed from the Tenax^®^GR-column using a Unity series 2 Thermal Desorption system (Markes, Llantrisant, UK) by heating the tube to 260°C (10 min at 20ml.min^-1^). Prior to desorption, tubes were dry purged for 4 minutes at 20ml.min^-1^. Next, analytes were refocused on a microtrap filled with Tenax^®^TA, cooled at -10°C. After flash-heating the microtrap at 280°C for 3 min, analytes were carried by a He-flow (constant pressure: 50 kPa) and injected with a split-flow of 5 ml.min^-1^ onto a 30m FactorFour VF-1ms low-bleed bounded-phase capillary GC-column (Varian, Sint-Katelijne-Waver, Belgium; 100% polydimethylsiloxane, internal diameter 0.25mm, film thickness 1mm). The flow path was heated to 130°C. The GC (Focus GC, Thermo Finnigan, Milan, Italy) oven temperature was initially set at 35°C for and kept for 10 minutes, then heated to 60°C at a rate of 2°C.min^-1^. Afterwards the temperature was increased to 170°C at 8°C.min^-1^ and finally to 240°C (at 15°C.min^-1^), maintained for 10 min. The MS transfer line was heated to 240°C. The ion source was put at 220°C. Masses with m/z 29 to 300 were recorded in full scan mode (200 ms/scan) on a DSQII Single Quadrupole MS (Thermo Finnigan, Austin, TX, USA), hyphenated to the GC, and operating at an electron impact energy of 70 eV. Chromatograms and mass spectra were processed using XCalibur software (Thermo Finnigan, v2·2) and the NIST database. For unidentified compounds, the Kováts retention index (I_*K*_) was calculated.

### Electronic nose (eNose) analysis

Exhaled VOCs were thermally desorbed from Tenax^®^GR tubes using nitrogen as carrier gas. Next, samples were analyzed by an assembly of four different eNoses, based on deviant sensor technologies: Cyranose C320 [26], Tor Vergata eNose [27], Common Invent eNose [28], and Owlstone Lonestar [29]. When exposed to a gas mixture, the sensors swell, resulting in a change of electrical resistances (ΔR). The ΔR/R-values are stored as raw data, producing a breathprint that describes the VOC mixture which can be used for pattern-recognition algorithms [30, 31]. The final eNose-based breath profiles were established by merging the sensor defections of all four devices.

### Statistics

R (v3.3.1) using the R studio interface was used for data analysis. Categorical variables are compared using a Pearson Chi^2^-test and reported as ratios. For continuous variables, normality was checked by a Shapiro-Wilk test. Dependent on the outcome, variables are given as mean (standard deviation) or median (quartile 1-quartile 3).

The raw eNose data were reduced by principle components analysis into principle components (PC). PCs explaining at least 70% of variance were retained and subsequently used as independent variables for linear discriminant analysis. The leave-one-out cross-validated (LOOCV) accuracy was reported in order to limit false discoveries. Receiver operating characteristic (ROC) curves were constructed.

For GC-MS data, the high number of variables and the rather low number of samples requires penalized logistic regression using the least absolute shrinkage and selection operator (lasso) to search for VOCs that have the most discriminative power for distinguishing MPM patients from controls. We used the *glmnet* R-package (v2.0-2) for fitting binomial lasso logistic models. This involves the selection of a tuning parameter (λ) that determines the number of selected VOCs. The optimal λ is selected by fitting the model for a sequence of λ-values, and for each of the λ-values the fitted model is evaluated by estimating the misclassification error rate by LOOCV. The λ-value minimizing this error rate was selected and used to fit the final model. Using the predicted outcomes of all of the patients, we then constructed an ROC curve (using the *ROCR* R-package (v1.0-7)) and estimated sensitivity, specificity, positive (PPV) and negative predictive value (NPV), the diagnostic accuracy of the final model, and the area under the curve (AUC_ROC_) with their 95% confidence intervals. We furthermore examined the number of times a VOC was selected by the lasso regressions. Variables selected in >50% of folds were considered important.

## CONCLUSION

GC-MS and eNose analysis allowed to discriminate MPM persons from asymptomatic, former asbestos-exposed persons at risk for MPM with great accuracy. The VOCs diethyl ether, methylcyclopentane, nonanal, limonene, cyclohexane, VOC I_*K*_ 1287 and isothiocyanatocyclohexane were identified as promising biomarkers for MPM. These data provide the proof-of-principle for future screening of persons at risk for MPM as a step-up tool in its diagnosis, making it less-invasive for the patient.

## SUPPLEMENTARY MATERIALS FIGURES


