# Supplementary material for: Breath analysis by gas chromatography-mass spectrometry and electronic nose to screen for pleural mesothelioma: a cross-sectional case-control study
Source: Oncotarget. 2017 Sep 27;8(53):91593–602. doi: 10.18632/oncotarget.21335 (PMC5710949; doi:10.18632/oncotarget.21335)
Supplement: Supplementary file 1 [file oncotarget-08-91593-s001.pdf]

Breath analysis by gas chromatography-mass spectrometry and electronic nose to screen for pleural mesothelioma: a cross-sectional case-control study

SUPPLEMENTARY MATERIALS

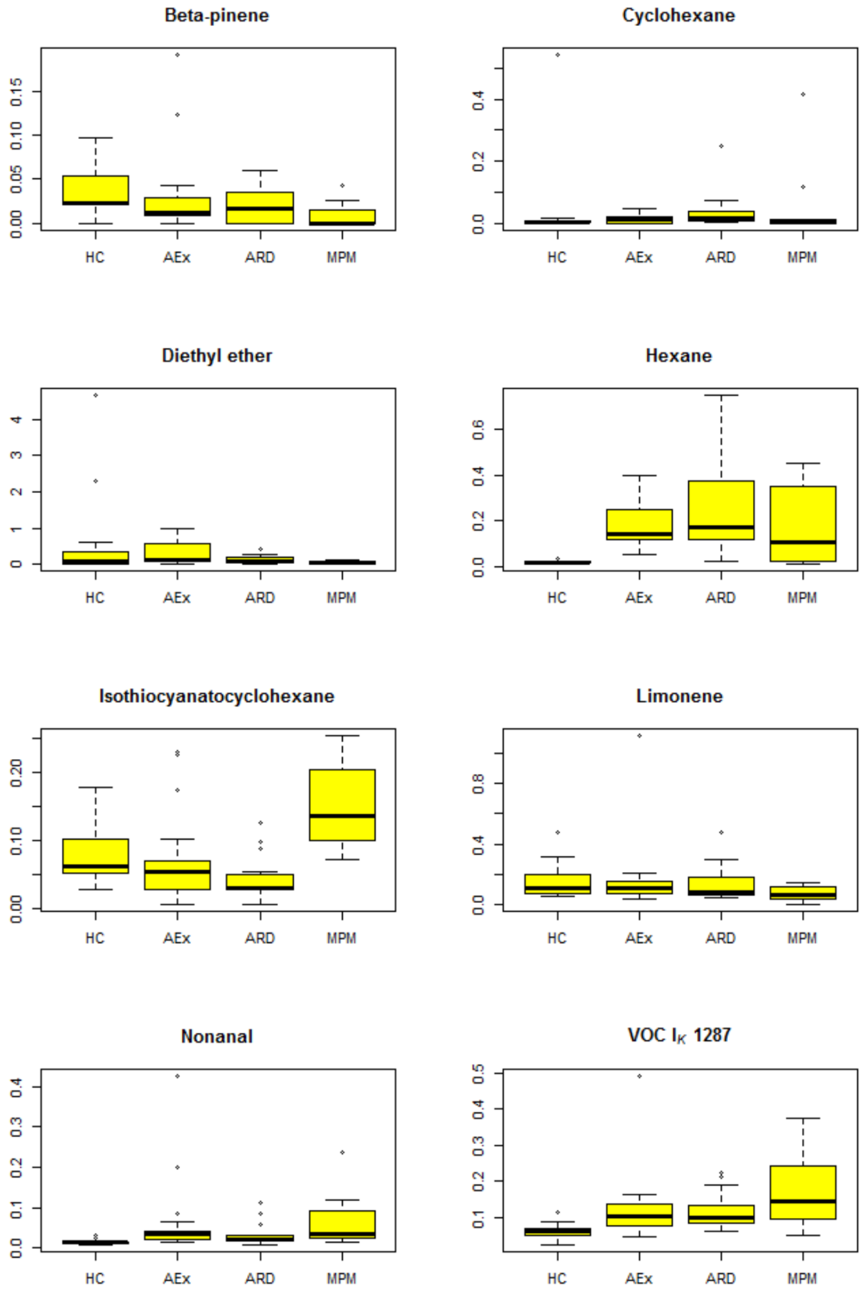

Supplementary Figure 1: Boxplots of important selected VOCs by lasso regression. AEx: asymptomatic former asbestos-exposed controls. ARD: patients with benign asbestos related diseases. HC: healthy controls. I_K: Kováts retention index. MPM: malignant pleural mesothelioma. VOC: volatile organic compound.

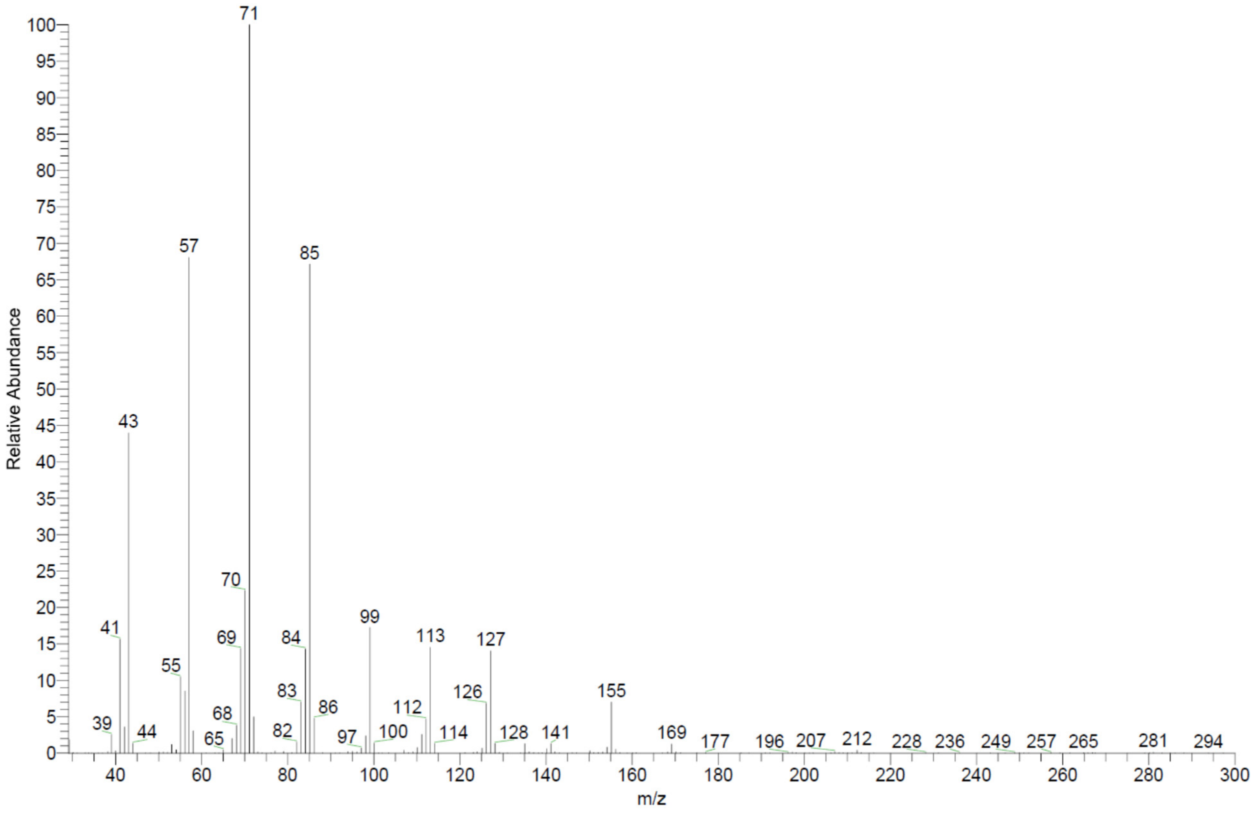

Supplementary Figure 2: Mass spectrum of VOC I_K 1287.
